# Next-Generation Sequencing in Pulmonary Fibrosis: Translational Promise and Current Clinical Limitations

**DOI:** 10.3390/cimb48070721

**Published:** 2026-07-15

**Authors:** Raffaella Pagliaro, Fabio Perrotta, Stefano Sanduzzi Zamparelli, Valerio Maria Carrozzo, Alfredo Cipriano, Michele Mondoni, Giulia Maria Stella, Andrea Bianco, Filippo Scialò

**Affiliations:** 1 Department of Translational Medical Sciences, University of Campania “Luigi Vanvitelli”, 81100 Caserta, Italy; raffaella.pagliaro@studenti.unicampania.it (R.P.); valerio.carrozzo96@gmail.com (V.M.C.); andrea.bianco@unicampania.it (A.B.); 2 Clinic of Respiratory Diseases “Vanvitelli”, A.O. dei Colli, Monaldi Hospital, 80131 Naples, Italy; 3 Division of Pneumology and Semi-Intensive Respiratory Therapy, A. Cardarelli Hospital, 80131 Naples, Italy; stefano.sanduzzizamparelli@aocardarelli.it; 4 Department of Molecular Medicine and Medical Biotechnology, University of Naples Federico II, 80131 Naples, Italy; alfredo.cipriano@unina.it (A.C.); filippo.scialo@unina.it (F.S.); 5 CEINGE-Biotecnologie Avanzate Franco Salvatore, 80145 Naples, Italy; 6 Respiratory Unit, Department of Health Sciences, ASST Santi Paolo E Carlo, University of Milan, 20142 Milan, Italy; michele.mondoni@unimi.it; 7 Department of Internal Medicine and Medical Therapeutics, Medical School, University of Pavia, 27100 Pavia, Italy; g.stella@smatteo.pv.it; 8 Unit of Respiratory Diseases, Cardiothoracic and Vascular Department, IRCCS Policlinico San Matteo, 27100 Pavia, Italy

**Keywords:** pulmonary fibrosis, next-generation sequencing, whole-genome sequencing, RNA sequencing, personalised medicine

## Abstract

Pulmonary fibrosis (PF), particularly idiopathic pulmonary fibrosis (IPF), is a progressive and often fatal interstitial lung disease characterised by complex genetic and molecular heterogeneity. Traditional diagnostic approaches, which rely on clinical, radiological and histopathological assessment, are frequently insufficient to capture the underlying biological diversity of the disease. The advent of next-generation sequencing (NGS) has substantially advanced the understanding of PF by enabling comprehensive genomic and transcriptomic profiling. NGS technologies, including whole-exome sequencing (WES), whole-genome sequencing (WGS), RNA sequencing (RNA-seq), and targeted gene panels, have uncovered key genetic determinants. These include mutations in telomere-related genes (TERT, TERC, RTEL1) and surfactant-related genes (SFTPC, SFTPA2), as well as common variants like the MUC5B promoter polymorphism. These discoveries have clarified disease pathogenesis, revealed polygenic risk models, and may improve diagnostic accuracy, particularly in distinguishing overlapping interstitial lung disease (ILD) phenotypes. Beyond genetics, transcriptomic analyses have identified dysregulated pathways, including TGF-β, Wnt/β-catenin, and PI3K/Akt signalling, and have enabled the discovery of novel biomarkers for prognosis and therapeutic response. In selected clinical settings, NGS is beginning to support patient stratification and inform management decisions. Emerging applications, including liquid biopsy and integration with artificial intelligence, further expand the potential clinical utility of NGS. Despite challenges related to cost, data interpretation and standardisation, NGS represents a powerful research tool in PF.

## 1. Introduction

Pulmonary fibrosis (PF) encompasses a heterogeneous group of interstitial lung diseases (ILDs) characterised by progressive scarring of the lung parenchyma, declining lung function and increased mortality. It is important to clarify that PF is a broad clinical and radiological descriptor encompassing multiple fibrotic interstitial lung diseases (e.g., idiopathic pulmonary fibrosis (IPF), connective tissue disease-associated ILD, chronic hypersensitivity pneumonitis, and familial forms). In contrast, usual interstitial pneumonia (UIP) refers to a specific histopathological and high-resolution CT pattern that defines IPF. Throughout this review, we use PF to denote the overarching fibrotic disease spectrum, while UIP is reserved for the specific imaging/histological pattern. IPF is the most common idiopathic fibrotic ILD and carries a poor prognosis, with median survival commonly reported between 3 and 5 years after diagnosis [1]. Despite advances in understanding, the translation of genomic discoveries into improved patient outcomes has not yet been realised. Most NGS applications in PF remain within the research domain. Pathobiologically, PF reflects aberrant epithelial injury and repair, fibroblast activation and excessive extracellular matrix deposition, leading to architectural distortion and respiratory failure [2,3]. Diagnostic classification remains challenging despite major advances in imaging and multidisciplinary assessment. Current frameworks rely on multidisciplinary discussion integrating high-resolution computed tomography (HRCT), clinical features and, where available, histopathology [4]. Overlapping phenotypes among ILDs—such as chronic hypersensitivity pneumonitis (cHP) and connective tissue disease-associated ILD (CTD-ILD)—often complicate accurate diagnosis and delay appropriate management. Importantly, wherever NGS findings are phenotype-dependent, we explicitly distinguish between specific ILD subtypes (IPF, CTD-ILD, FPF, HP, etc.) throughout this review. Genetic and molecular insights have shown that fibrotic ILDs are defined not only by radiological or histological patterns but also by underlying biological mechanisms [5]. Familial pulmonary fibrosis (FPF) is reported in up to ~20% of idiopathic interstitial pneumonias, underscoring the contribution of heritable factors [6]. Early genetic studies identified mutations in surfactant-related genes (SFTPC, SFTPA2) and telomere-related genes (TERT, TERC), implicating epithelial dysfunction and telomere shortening as central mechanisms [7]. Genome-wide association studies identified common susceptibility variants, most notably the MUC5B promoter polymorphism (rs35705950), which confers increased IPF risk but shows incomplete penetrance [8]. Traditional Sanger sequencing helped identify these early associations but is limited by low throughput. NGS technologies have overcome this, enabling rapid, high-throughput analysis of genomes, exomes, and transcriptomes [9,10]. These advances have facilitated identification of rare pathogenic variants and common polymorphisms, supporting a polygenic model of disease risk. Beyond genomics, transcriptomic profiling via RNA-seq has revealed cell-specific gene expression patterns and dysregulated pathways in IPF lungs [11,12]. Single-cell RNA sequencing has uncovered previously unrecognised cell populations, such as aberrant basaloid epithelial cells [13]. The clinical utility of NGS differs by application: targeted germline testing may inform counselling in selected patients, whereas transcriptomics and AI integration remain translational tools. In this review, we outline NGS technologies and summarise their application to PF, distinguishing what is ready for clinical practice from what requires further validation.

## 2. Principles and Technologies of Next-Generation Sequencing

NGS refers to high-throughput methods that sequence millions of DNA or RNA fragments in parallel, enabling genome-wide or targeted interrogation of genetic variation and gene expression at scale. Compared with Sanger sequencing, NGS offers higher throughput and lower cost per base, allowing assessment of multiple genes in a single assay [9,14]. In PF, common approaches include whole-genome sequencing (WGS), whole-exome sequencing (WES), targeted gene panels, and RNA-seq. WGS captures coding and non-coding regions but is limited by cost and interpretation challenges [15]. WES targets protein-coding regions, offering a practical balance for identifying rare variants [16,17]. Targeted panels restrict sequencing to a curated set of genes (e.g., TERT, SFTPC, ABCA3), improving turnaround time and interpretability—a pragmatic first-line option in specialised ILD clinics. RNA-seq provides insights into gene expression and dysregulated pathways [17,18,19]. NGS presents challenges including data interpretation complexity, variants of uncertain significance (VUS), and the need for robust bioinformatics pipelines [20]. Outside specialised centres and research protocols, routine NGS for PF is not standard of care, and clinical decisions should not be based on unvalidated genetic findings. Table 1 provides a comparative overview of these technologies, highlighting advantages, limitations, relative costs, clinical applicability, and estimated diagnostic yield in PF.

## 3. Genetics of Pulmonary Fibrosis: Contributions from NGS

Family history assessment is an essential component of ILD evaluation. A positive family history is not only associated with an increased risk of IPF but also portends a worse prognosis, with affected individuals often experiencing earlier disease onset and more rapid progression [21]. FPF account for approximately 20% of idiopathic interstitial pneumonias, though this figure may underestimate the true heritable burden, as small family clusters, reduced penetrance, and incomplete family histories often obscure genetic transmission [22]. The advent of NGS has dramatically accelerated the discovery of both rare pathogenic variants and common susceptibility alleles that underpin fibrotic lung disease, transforming the genetic landscape from a handful of candidate genes to a complex polygenic architecture. Table 2 provides a comprehensive summary of the major genetic contributors identified to date, categorised by biological pathway, variant type, and mechanistic role.

### 3.1. Telomere-Related Genes and the Senescence Paradigm

Telomere maintenance has emerged as a central mechanistic pathway in PF. Telomeres, the repetitive nucleotide sequences at chromosome ends, protect genomic integrity by preventing DNA degradation and end-to-end fusion. With each cell division, telomeres shorten progressively until a critical threshold is reached, triggering DNA damage responses and cellular senescence—a process known as the Hayflick limit. In the lung, alveolar type II (ATII) epithelial cells serve as resident progenitor cells responsible for epithelial repair following injury; their replicative capacity is therefore tightly linked to telomere length. Rare pathogenic variants in telomere-related genes—including TERT, TERC, RTEL1, PARN, TINF2, and DKC1—impair telomerase function or telomere maintenance, leading to accelerated telomere attrition and premature cellular senescence [23,24,25]. The resulting impairment of ATII regenerative capacity renders the lung vulnerable to repetitive micro-injuries, aberrant wound healing, and ultimately progressive fibrosis. Importantly, telomere-related variants are not confined to the lung; affected patients may present with systemic short-telomere syndromes such as dyskeratosis congenita, characterised by reticulated skin pigmentation, nail dystrophy, oral leukoplakia, bone marrow failure, hepatic cirrhosis, and early-onset malignancies [26,27]. The pleiotropic nature of these variants underscores the importance of a comprehensive, multi-system clinical assessment when interpreting NGS findings. Mechanistically, telomere shortening activates the p53/p21 pathway, promoting cellular senescence and the senescence-associated secretory phenotype (SASP), which in turn perpetuates chronic inflammation and fibroblast activation through paracrine signalling [25]. The biological centrality of telomere dysfunction has also spurred interest in telomere-targeted therapeutic strategies, including telomerase activators and senolytic agents, though these remain investigational.

### 3.2. Surfactant-Related Genes and Alveolar Type II Epithelial Dysfunction

Pulmonary surfactant, produced exclusively by ATII pneumocytes, serves dual roles in reducing alveolar surface tension (thereby preventing atelectasis) and supporting local innate immune defence through opsonisation and phagocytosis enhancement [28]. Genetic disruption of surfactant homeostasis directly implicates ATII epithelial dysfunction as a critical driver of fibrosis.

Rare variants in SFTPC (surfactant protein C) are typically inherited in an autosomal dominant manner with variable penetrance. The most common disease-associated mutation, the SFTPC I73T variant, results in misfolded pro-SP-C protein that accumulates in the endoplasmic reticulum (ER) of ATII cells, triggering the unfolded protein response (UPR), ER stress, and ultimately ATII apoptosis [29]. This ER stress pathway is now recognised as a key upstream event in fibrotic lung disease, linking genetic susceptibility to cellular injury and aberrant repair. ABCA3 (ATP-binding cassette transporter A3) encodes a lipid transporter critical for lamellar body formation and surfactant packaging; biallelic loss-of-function variants typically present as severe neonatal or childhood interstitial lung disease (chILD), though compound heterozygous or mild hypomorphic variants can occasionally manifest as adult-onset progressive fibrosis [29]. SFTPA1 and SFTPA2 encode collectin proteins that participate in innate immune defence; their variants are rarer but have been mechanistically linked to ATII dysfunction through impaired immune surveillance and altered surfactant homeostasis, potentially creating a permissive environment for both fibrotic remodelling and epithelial injury [29]. Collectively, the surfactant-related genes underscore a paradigm in which genetic lesions affecting ATII cell homeostasis—whether through protein misfolding, lipid trafficking defects, or immune dysregulation—create a permissive environment for fibrotic remodelling.

### 3.3. Common Susceptibility Loci and the Polygenic Architecture

Beyond rare Mendelian variants, GWAS have revolutionised understanding of pulmonary fibrosis as a polygenic disease, wherein the cumulative burden of multiple common alleles, each with modest effect sizes, modulates individual susceptibility. The most robust and replicated finding is the MUC5B promoter polymorphism (rs35705950), a common variant that confers the strongest genetic risk for both sporadic and familial IPF [8,30]. MUC5B encodes a gel-forming mucin that is normally expressed in proximal airways but is aberrantly upregulated in the distal lung, particularly within honeycomb cysts and metaplastic bronchiolar epithelium, in carriers of the risk allele. The pathophysiological mechanism remains incompletely understood, but increased MUC5B expression in the alveolar niche may impair mucociliary clearance of inhaled pathogens and particulates, creating a microenvironment of chronic low-grade inflammation and repetitive epithelial injury that predisposes to fibrosis. Alternatively, MUC5B overexpression may directly influence epithelial cell behaviour and wound healing responses. Additional GWAS loci have expanded the genetic architecture. TOLLIP (Toll-interacting protein) encodes a negative regulator of Toll-like receptor (TLR) signalling, implicating innate immune dysregulation in disease pathogenesis. DSP (desmoplakin) is a component of desmosomes, cell–cell adhesion junctions critical for epithelial integrity; its association with IPF suggests that impaired epithelial barrier function may contribute to disease susceptibility. FAM13A (family with sequence similarity 13 member A) is involved in cell cycle regulation and β-catenin signalling, while OBFC1 (oligonucleotide/oligosaccharide-binding fold containing 1) participates in telomere maintenance, providing a mechanistic link between common variation and the telomere senescence pathway [30]. Together, these loci support a convergent biological model in which epithelial injury, immune activation, telomere dysfunction, and impaired tissue repair collectively determine an individual’s risk of developing pulmonary fibrosis.

### 3.4. Interstitial Lung Abnormalities and Preclinical Genetic Risk

Interstitial lung abnormalities (ILAs), defined as incidental findings of parenchymal changes on chest CT in individuals without suspected ILD, represent a preclinical reservoir that may progress to overt pulmonary fibrosis. Genetic factors, particularly the MUC5B variant and telomere shortening, are consistently associated with ILA presence, extent, and progression [8,30]. This observation positions genetic susceptibility as an early, potentially preclinical determinant of disease trajectory. However, the utility of common variants in screening or risk stratification remains constrained by incomplete penetrance, variable expressivity, and the absence of validated polygenic risk scores (PRS) that perform consistently across diverse populations. Emerging efforts to integrate multiple GWAS loci into PRS for IPF have shown promise in retrospective cohorts, but prospective validation, particularly across different ancestries, is lacking.

### 3.5. Missing Heritability and Gene-Environment Interactions

Despite substantial progress, a considerable portion of the heritability of pulmonary fibrosis remains unexplained—a phenomenon often termed “missing heritability.” This gap likely reflects the combined effects of: (i) rare variants in genes not yet discovered or not captured by current platforms (e.g., complex structural rearrangements, repeat expansions, or regulatory variants in non-coding regions); (ii) synergistic interactions between multiple rare and common variants; (iii) epigenetic modifications that modulate gene expression without altering the underlying DNA sequence; and (iv) environmental and lifestyle factors (e.g., smoking, occupational dust exposure, viral infections, and gastro-oesophageal reflux) that interact with genetic predisposition to trigger or accelerate disease. NGS technologies, particularly whole-genome sequencing (WGS) and integrated multi-omics approaches, hold promise for uncovering the missing heritability, but large-scale, well-phenotyped longitudinal cohorts are essential to disentangle these complex gene-environment interactions.

### 3.6. Ancestry Gaps and the Need for Global Diversity

A critical limitation of the current genetic landscape in pulmonary fibrosis is the striking predominance of European-ancestry cohorts in discovery and replication studies. The frequency of the MUC5B risk allele, for instance, differs substantially across racial and ethnic groups, and telomere length distributions show ancestry-dependent variation that may influence both disease risk and survival estimates [30,31]. These disparities not only limit the generalisability of existing genetic knowledge but also raise concerns about the equitable application of NGS-based testing in non-European populations. Future studies must prioritise the inclusion of diverse, multi-ethnic cohorts to ensure that genetic discoveries translate into clinically meaningful and globally applicable tools.

## 4. NGS in the Differential Diagnosis of Pulmonary Fibrosis

### 4.1. NGS as a Tool for Distinguishing Between Overlapping Fibrotic Phenotypes

NGS can support differential diagnosis, yet its diagnostic yield and clinical interpretation differ substantially between familial and sporadic forms of pulmonary fibrosis. To clarify these distinct roles, this subsection is divided accordingly.

#### 4.1.1. NGS in Familial Pulmonary Fibrosis (FPF)

In multicentre cohorts employing whole-exome sequencing (WES) or targeted panels, the diagnostic yield of NGS is highest in FPF. Pathogenic variants are identified in approximately 20–30% of familial cases, predominantly involving telomere-related genes (TERT, TERC, RTEL1, PARN, POT1, TINF2, DKC1). Surfactant-related genes (SFTPA1/2, SFTPC, ABCA3) account for a smaller proportion, detected in roughly 1–3% of FPF [32,33,34,35]. Identifying these inherited patterns enables the recognition of biologically defined subgroups, facilitating targeted genetic counselling, systematic extrapulmonary assessment (e.g., for occult telomere syndromes), and informed pre-transplant planning [6]. However, even in the familial setting, these findings primarily refine biological understanding and prognostic stratification rather than fundamentally reclassifying the underlying ILD diagnosis.

#### 4.1.2. NGS in Sporadic and Other Fibrotic ILDs

In contrast, the role of NGS in sporadic fibrotic ILDs is more constrained. Pathogenic variants are detected in only approximately 5–10% of such cases [25]. Common susceptibility alleles, such as the MUC5B promoter polymorphism (rs35705950), contribute to risk across multiple fibrotic phenotypes but lack standalone diagnostic specificity, given their appreciable prevalence in healthy controls and their variable ancestry-dependent frequencies [8,36,37,38,39]. Nevertheless, NGS retains clear diagnostic value in rare, highly penetrant entities with strong genetic determinism, such as heritable pulmonary veno-occlusive disease (biallelic EIF2AK4 variants) [36]. For typical sporadic IPF presenting with a usual interstitial pneumonia (UIP) pattern in an older patient, the likelihood of identifying an actionable monogenic variant is notably low. Consequently, NGS is most appropriately reserved for specific clinical scenarios where the pre-test probability of an inherited cause is elevated: young-onset disease, positive family history, extrapulmonary features of telomere syndromes, or atypical imaging patterns [6]. While transcriptomic and gene expression patterns offer future promise, they currently remain translational tools that complement—rather than replace—multidisciplinary assessment [40].

### 4.2. Integration with Clinical, Radiological, and Histological Data

**Figure 1:**
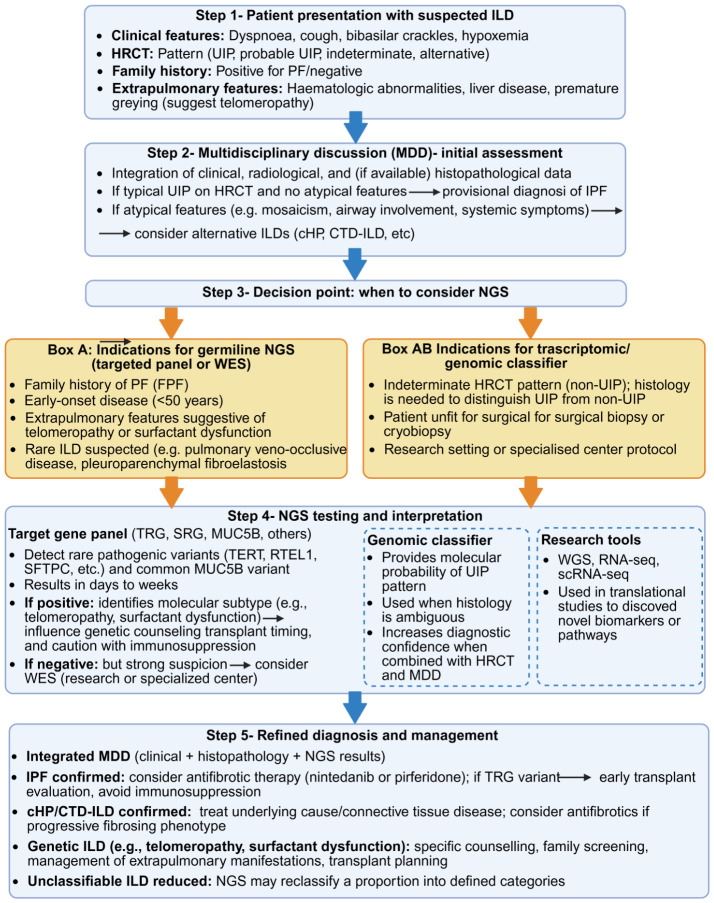
Clinical framework integrating NGS into diagnostic pathway for fibrotic ILD. Abbreviations: UIP= usual interstitial pneumonia; cHP= chronic hypersensitivity pneumonitis; CTD-ILD= connective tissue disease-associated interstitial lung disease; TRG= telomere-related gene; SRG= surfactant-related gene; TBLC= transbronchial lung cryobiopsy; SLB= surgical lung biopsy; WES= whole-exome sequencing. Dashed lines indicated that genomic classifier and WES are not universally available; their use varies by center.

In routine practice, thorough MDD integrating HRCT and clinical features remains the diagnostic gold standard, with NGS playing an adjunctive role in selected cases. By elucidating genetic underpinnings, NGS allows more precise subclassification, improving prognostic accuracy and fostering personalised strategies [32]. Figure 1 outlines a proposed pathway for integrating NGS into diagnostic evaluation.

The diagnostic yield of NGS is highest when interpreted in a multidisciplinary context integrating genomic, radiologic, and histopathologic data [41]. Incorporating variant reports, HRCT, and lung biopsy modifies diagnoses or treatment in up to 40% of ILD evaluations [42]. Genomic classifiers such as the Envisia™ Genomic Classifier (EGC) enhance diagnostic precision, distinguishing UIP from non-UIP patterns with 68% sensitivity and 92% specificity [43,44,45]. Although EGC performance is reduced in non-UIP HRCT patterns, it remains valuable in ambiguous cases, increasing IPF recognition rates to 89–93% [46,47]. When integrated with radiology, EGC enhances diagnostic sensitivity from 60.3% to 79.2% and shows 76% concordance with transbronchial lung cryobiopsy, providing definitive diagnoses in 94% of ILD cases [48]. In a gene-MDD cohort, targeted NGS identified pathogenic variants in 61% of FPF referrals, leading to revised diagnoses in one-third and treatment changes in 26% [42]. Emerging evidence supports artificial intelligence (AI) for integrating molecular and imaging data [49]. Integrating scRNA-seq with machine learning has identified specific inflammatory pathways as potential therapeutic targets in RA-ILD. AI-driven platforms have identified novel inhibitors of fibrotic pathways with preclinical and early-phase efficacy [50]. However, these integrations are early-stage; most evidence is preclinical or retrospective.

### 4.3. Use of Targeted Gene Panels for More Precise Diagnosis

Targeted gene panels enable simultaneous analysis of multiple ILD-associated genes. Current panels (3–300 genes) are cost-effective first-line tools in specialised ILD clinics for suspected FPF. The EGC, a targeted expression panel, may reduce the need for invasive procedures [51]. Several laboratory-developed tests exist, but composition is nonstandardised [52]. Modern ILD panels include emerging loci associated with PF and short-telomere syndromes, while retaining key variants like MUC5B rs35705950 for its prognostic value [53]. Panels also aid in detecting rare entities such as pleuroparenchymal fibroelastosis (PPFE), where telomere biology disorder variants are common [54]. Genetic insights guide management, as patients with telomere or surfactant abnormalities may have poorer outcomes and require tailored approaches to immunosuppression and radiation.

Targeted gene panels provide sensitive and specific first-line testing for many genetic disorders, yet their composition lacks standardisation due to genetic heterogeneity and ongoing gene discovery [55]. NGS-based testing for ILD remains limited by incomplete variant detection (repeat expansions, complex rearrangements) and panel coverage, resulting in low diagnostic yield [56]. Practical implementation also faces barriers including variable insurance reimbursement and inconsistent access to genetic counselling. Furthermore, technical heterogeneity across different commercially available and laboratory-developed NGS platforms—varying in target coverage depth, read length, and proficiency in GC-rich or repeat-rich regions—can influence the detection of complex structural variants and repeat expansions, adding a layer of variability to diagnostic yield that clinicians should be aware of when interpreting negative results.

## 5. Transcriptomics and Biomarker Discovery Through NGS

### 5.1. RNA-Seq and Gene Expression Profiling in Fibrotic Diseases

RNA-seq has emerged as a key tool for elucidating disease mechanisms via quantitative transcriptomic profiling at tissue and single-cell resolution [57]. Integrative single-cell and bulk RNA-seq have refined ILD classification, revealing immune infiltration and downregulation of ubiquitin-related genes, suggesting shared mechanisms across COPD, COVID-19, and ILDs [58,59,60]. In IPF, RNA-seq has identified dysregulated pathways in inflammation, fibrosis, and cellular heterogeneity [61]. Multi-omics approaches integrating transcriptome-wide association studies (TWAS) and GWAS have identified IPF-associated genes that promote fibrosis through chronic inflammation, impaired silencing of pro-inflammatory genes, or epithelial–mesenchymal transition [62]. RNA-seq has delineated cell type-specific transcriptional signatures. In alveolar type II cells, certain genes are dysregulated; mesenchymal cells show altered expression of genes involved in fibroblast activation and ECM deposition [63,64]. Even “normal-appearing” lung regions in IPF exhibit transcriptional activation of fibrotic and immune-related genes [65]. RNA-seq also has prognostic potential. Bulk RNA-seq of IPF explants has identified enrichment of pathways related to T-cell activation, tumorigenesis, and lung function decline [66]. Reduced expression of certain genes correlates with fibroblast inhibition. In advanced disease, immune and fibrotic pathways are upregulated, including immune checkpoint molecules and chemokine axes [67]. Gene expression correlates with FVC decline [68], and studies have identified markers of disease progression including proteins and microRNAs [69,70,71]. Unsupervised clustering of RNA-seq data has revealed distinct cellular subsets in IPF: a myeloid-enriched cluster with elevated fibrotic signatures and a ciliated epithelial-dominant cluster associated with B-cell/plasma cell signatures and poorer prognosis [72].

### 5.2. Identification of Key Dysregulated Pathways

RNA-seq has advanced understanding of ILD cellular and molecular complexity, revealing marked transcriptional divergence between healthy and fibrotic lungs [63]. Multi-omics approaches have identified dysregulated pathways including mTOR signalling, DNA damage response, and innate immune activation [73]. Hundreds of differentially expressed genes are involved in ECM remodelling and immune regulation [74]. Gene expression profiling has revealed epithelial dysfunction and immune activation in IPF. Upregulated genes are associated with epithelial stress and innate immunity; downregulated genes with transcriptional regulation and ECM homeostasis [75]. Dysregulated fibroblast-associated genes implicate altered proliferation and matrix remodelling, with miRNA-mediated regulatory loops [76,77,78]. WES in diverse populations has identified deleterious variants in genes involved in immune regulation, epithelial barrier integrity, intracellular trafficking, and tissue remodelling [79]. GWAS confirmed contributions of host defence and cell adhesion genes [30,80]. Oncogenic mutations have been identified in non-neoplastic IPF tissue, suggesting overlap between fibrosis and malignancy [81]. Proteomic analyses show elevated profibrotic markers correlating with disease severity. RNA-seq has clarified cellular heterogeneity: distinct ATII cell populations express fibrosis-related genes with maladaptive Wnt signalling, and impaired ATII-to-ATI differentiation disrupts epithelial homeostasis [82]. Basal cell expansion includes subsets associated with poor prognosis and ECM production [83]. Single-cell analyses identified aberrant basaloid cells co-expressing epithelial and mesenchymal markers [13]. The stromal compartment shows marked heterogeneity, with myofibroblasts driving ECM deposition [63]. Immune cells play a central role, with specific macrophage subsets promoting fibrosis [84,85,86], including transitional profibrotic macrophages [87,88]. Endothelial remodelling with ectopic cell populations reflects aberrant angiogenesis [89].

### 5.3. Discovery of Molecular Phenotypes and Potential Biomarkers for Prognosis or Treatment Response

NGS provides quantitative prognostic insights. Rare telomere-related variants (TERT, RTEL1, PARN) are associated with accelerated FVC decline, reduced transplant-free survival, and increased risk of lung cancer and liver dysfunction. TRG polymorphisms predict adverse outcomes and poor response to immunosuppression. The TOLLIP rs5743890 variant reduces IPF susceptibility but paradoxically increases mortality [90]. Surfactant-related mutations (SFTPA1/2) increase lung cancer risk independently [91]. The MUC5B rs35705950 variant increases IPF risk >3-fold but is associated with later onset, slower progression, and improved survival in some studies [8,92]. It is also linked to other fibrotic ILDs with later onset [93,94]. Absence of the MUC5B variant correlates with increased ILA incidence and progression [95,96]. GWAS have identified additional prognostic variants associated with faster FVC decline and increased mortality [97,98]. Transcriptomic signatures and multiomic classifiers stratify patients by progression-free survival [99,100]. Metagenomic NGS has utility in acute exacerbations of ILD, identifying microbial burden [101,102].

### 5.4. Pharmacogenomic Insight and Its Limitations

Genetic variants may influence response to antifibrotic therapies. Polymorphisms in TOLLIP, TGF-β1, and DSP have been associated with differential responses to nintedanib and pirfenidone [103,104,105]. Nintedanib downregulates genes involved in the cell cycle, angiogenesis, and inflammation [106], modulating MAPK, PI3K/AKT, Wnt/β-catenin, and TGF-β pathways [107,108]. Pirfenidone slows FVC decline regardless of TRG or MUC5B genotype [109,110], modulates TGF-β and Wnt pathways, and downregulates profibrotic genes [111,112]; however, in SSc-ILD, it may enhance inflammatory signalling [113]. Genetic insights are guiding targeted therapies: in ABCA3 deficiency, certain agents may restore surfactant function [114]; precision approaches include sirolimus and sotatercept [115]; and targeting MUC5B overexpression is promising [116]. However, these pharmacogenomic associations derive from underpowered, retrospective studies without prospective validation. No major guideline recommends pharmacogenomic testing to guide antifibrotic therapy in PF.

## 6. Impact of NGS on Therapy and Precision Medicine

The influence of NGS on PF therapy is still evolving. No prospective trial has demonstrated that NGS-guided management improves patient outcomes. Genomic stratification may inform family counselling and transplant decisions in FPF, but for sporadic IPF, NGS does not currently guide the choice of or predict response to approved antifibrotic therapies [35]. To help readers understand which NGS platform is most suitable for a given clinical or research question, Table 3 summarises the utility of different NGS technologies (targeted panels, WES, WGS, RNA-seq, scRNA-seq) for PF/IPF detection, highlighting their advantages, limitations, cost, diagnostic yield, and clinical status. Telomere-related variants are associated with more aggressive disease and may warrant early transplant referral [110]. Pharmacogenomic associations (TOLLIP, TGF-β) require prospective validation [117,118]. NGS has facilitated identification of novel therapeutic targets (Wnt/β-catenin, PI3K/Akt, mTOR). It may also enable molecular stratification in clinical trials to increase efficiency. Moreover, Table 4 provides a curated list of key published NGS and GWAS studies in pulmonary fibrosis, including large-scale, high-impact cohorts. For each study, the table reports the design, sample size, sequencing platform, main genetic or transcriptomic findings, clinical relevance, and methodological limitations—offering a concise evidence map for researchers and clinicians. For clinicians managing suspected sporadic IPF, NGS is not indicated for routine use, as it provides no proven benefit for first-line therapy selection.

## 7. Translational Potential and Clinical Limitations of NGS in PF

Despite significant research contributions, translating NGS into routine PF practice faces well-defined limitations.

### 7.1. What NGS Can Currently Offer

(1) Genetic diagnosis in suspected FPF (diagnostic yield 20–30% for telomere variants) [35].
(2) Identification of pathogenic variants in early-onset ILD (<50 years) or with telomere syndrome features.
(3) Guidance for family counselling and early transplant referral in telomere variant carriers.
(4) Definitive diagnosis of rare monogenic ILDs (e.g., EIF2AK4 in pulmonary veno-occlusive disease).

### 7.2. What NGS Cannot Yet Do

(1) Guide first-line choice between pirfenidone and nintedanib in sporadic IPF (no prospective pharmacogenomic validation).
(2) Replace MDD as diagnostic gold standard.
(3) Provide reliable individual prognostic stratification using common variants (e.g., MUC5B).
(4) Serve as a standalone screening test for ILAs or early IPF.

### 7.3. Key Clinical Limitations

(1) VUS: Substantial proportion, causing uncertainty and anxiety.
(2) Lack of standardisation: Panels vary widely; no universal panel.
(3) Cost and accessibility: WES/WGS expensive; inconsistent reimbursement.
(4) Genetic counselling gap: Many ILD clinics lack counsellors.
(5) Ancestry bias: Most data from European cohorts.

## 8. Practical Clinical Relevance: How NGS Informs Diagnosis, Prognosis, and Treatment

Connecting molecular findings to bedside practice requires clear, actionable guidance. Below we summarise the current clinical relevance of NGS in PF across three domains.

### 8.1. Diagnosis

NGS is most useful when clinical features suggest a genetic aetiology: young age, family history, atypical imaging (upper lobe predominance, PPFE pattern), or telomere syndrome stigmata. In such patients, a targeted panel can confirm FPF, distinguish it from other ILDs, and avoid unnecessary immunosuppression (which may harm telomopathy patients). In typical sporadic IPF, NGS has very low yield and does not alter diagnosis; routine testing is not indicated.

### 8.2. Prognosis

Telomere-related pathogenic variants predict faster FVC decline, shorter transplant-free survival, and higher likelihood of extrapulmonary complications (e.g., lung cancer, liver dysfunction). Surfactant-related gene variants (SFTPA1/2) are associated with increased lung cancer risk and may influence surveillance strategies. MUC5B rs35705950 carrier status is associated with slower disease progression in most studies, but this finding is not sufficiently robust for individual prognostic use. Transcriptomic signatures (e.g., 52-gene blood signature) remain research tools and are not yet clinically available.

### 8.3. Treatment Implications (Current and Emerging)

No guideline recommends using NGS to choose between pirfenidone and nintedanib in sporadic IPF. Both are approved regardless of genotype. For telomere variant carriers, antifibrotic therapy appears safe and may slow progression, but no randomised trial has specifically examined this subgroup [109]. Immunosuppressive agents (e.g., corticosteroids, mycophenolate) should be used with caution in telomere-related variant carriers, as they may accelerate decline [35]. Genetic findings may inform transplant referral: telomere variant carriers often have worse post-transplant outcomes and may require modified conditioning. Emerging targeted therapies (TNIK inhibitors, sirolimus, MUC5B-directed agents) are in early development; future NGS-based stratification may become relevant.

### 8.4. Current Guidelines and Expert Statements

PFF Genetic Testing Work Group (2022): Recommends targeted NGS panels for suspected FPF, early-onset ILD, and telomere syndrome features; advises against routine testing in sporadic IPF [35]. ERS statement on familial pulmonary fibrosis (2023): Supports genetic testing in FPF, emphasising counselling and multidisciplinary interpretation [52]. ATS/ERS/JRS/ALAT IPF guideline (2022): Does not recommend routine genetic testing for diagnosis but acknowledges its role in selected cases [45].

In summary, NGS has a circumscribed but valuable role in PF clinical practice, limited to specific patient subsets. Widespread routine use awaits prospective validation of pharmacogenomic markers and better standardisation.

## 9. Conclusions: Current Limitations and Future Perspectives

Despite its significant research impact, the implementation of NGS in PF faces several challenges. A primary limitation is the complexity of data interpretation, particularly VUS. Lack of standardisation across platforms and panels leads to variability in diagnostic yield. High costs and limited accessibility restrict widespread adoption, especially in resource-limited settings. Ethical considerations, including genetic counselling and management of incidental findings, further complicate integration. Looking forward, advances in multi-omics integration, AI, and machine learning are expected to enhance data interpretation. Emerging approaches such as liquid biopsy and single-cell sequencing may enable real-time disease monitoring. A key priority for the field is to move from discovery-oriented association studies to interventional trials that test whether incorporating genomic information improves patient outcomes. Based on current guidelines and expert consensus, practical indications for considering NGS testing in PF include: suspected familial PF (≥2 family members with ILD); early-onset ILD (<50 years); and extrapulmonary features suggestive of a telomere syndrome. Conversely, routine NGS testing is not currently recommended for patients with sporadic, typical IPF presenting after age 60 years with a definite UIP pattern and no family history. However, this recommendation may need to be revisited as costs decrease, evidence accumulates, and pharmacogenomic markers potentially become available to guide differential therapy response.

## Figures and Tables

**Table 1:** Comparative overview of next-generation sequencing technologies in pulmonary fibrosis. Advantages, limitations, relative cost, estimated diagnostic yield, and current method of detection for each platform.

Technology	Detection	Advantages	Limitations	Relative Cost	Diagnostic Yield in PF (Estimated)
Targeted Gene Panels	Predefined set of genes (e.g., TERT, SFTPC, MUC5B)	Cost-effective; rapid turnaround; high depth of coverage; easier interpretation	Limited to known genes; may miss novel variants	Low ($)	Sporadic: ~5–10%; Familial: ~20–30%
Whole-Exome Sequencing (WES)	Protein-coding regions	Broader coverage than panels; can identify novel genes	Higher cost; incidental findings; interpretation challenges	Moderate ($$)	~10–20% (higher in FPF)
Whole-Genome Sequencing (WGS)	Entire genome (coding and non-coding)	Most comprehensive; detects non-coding and structural variants	Highest cost; data storage/analysis complexity	High ($$$)	Similar to WES plus structural variants
RNA Sequencing (RNA-seq)	Gene expression levels; splicing; non-coding RNAs	Functional insights; can validate genomic findings; reveals cellular heterogeneity	Requires high-quality RNA; complex analysis; not standardised	Moderate ($$)	Not applicable (research classifier)
Single-cell RNA-seq (scRNA-seq)	Gene expression at individual cell resolution	Highest resolution of tissue biology	Very high cost; complex analysis; difficult to standardise	Very high ($$$$)	Not applicable (discovery)

$ Relative cost: Low ($); Moderate ($$); High ($$$); Very high ($$$$).

**Table 2:** Key genetic contributors to pulmonary fibrosis identified through NGS.

Gene/Locus	Protein/Function	Variant Type	Frequency in FPF	Frequency in Sporadic ILD	Associated Phenotype/Syndrome	Clinical Actionability/Notes
Telomere-Related Genes						
*TERT*	Telomerase reverse transcriptase	Rare LoF/dominant negative	~15–20%	~1–3%	IPF, short-telomere syndromes (dyskeratosis congenita), hepatic cirrhosis	Inform transplant planning (risk of myelosuppression); genetic counselling
*TERC*	Telomerase RNA component	Rare LoF	~5–10%	<1%	IPF, short-telomere syndromes	As above; bone marrow failure screening
*RTEL1*	DNA helicase	Rare LoF	~2–5%	<1%	IPF, Hoyeraal-Hreidarsson syndrome	Associated with early onset/severe disease
*PARN*	Poly(A)-specific ribonuclease	Rare LoF	~2–4%	<1%	IPF, short-telomere syndromes	As above
*TINF2/DKC1*	Shelterin complex/Dyskerin	Rare LoF	<2%	<1%	Dyskeratosis congenita, severe short-telomere syndromes	Usually paediatric onset; rare in adult PF
Surfactant-Related Genes						
*SFTPC*	Surfactant protein C	Rare missense/dominant	~1–2%	<1%	Familial IPF, childhood ILD, adult fibrosis	Associated with atypical imaging (e.g., NSIP, CPFE)
*SFTPA1/SFTPA2*	Surfactant protein A1/A2	Rare missense	<1%	<1%	IPF, lung adenocarcinoma	Variants may disrupt innate immunity
*ABCA3*	ATP-binding cassette transporter	Rare biallelic LoF	<1%	<1%	Childhood ILD, adult PF	Typically severe early onset; rare in adult sporadic PF
High-Penetrance Rare Entities						
*EIF2AK4*	Eukaryotic translation initiation factor	Biallelic LoF	N/A	N/A	Pulmonary veno-occlusive disease (PVOD)	Diagnostic; confirms PVOD; alters transplant urgency
Common Susceptibility Loci						
*MUC5B* (rs35705950)	Mucin 5B (promoter variant)	Common SNP (T allele)	~30–40% (carrier)	~15–25% (carrier)	IPF, familial PF, chronic HP	Major risk factor; NOT diagnostic (present in healthy controls); useful for risk stratification in research
*Other GWAS loci* (e.g., *DSP, FAM13A*, *TOLLIP*, *OBFC1*)	Various (cell adhesion, telomere maintenance)	Common SNPs	Variable	Variable	Modest risk for IPF/ILD	Currently limited standalone clinical utility; polygenic risk scores unvalidated

**Table 3:** Comparison of NGS technologies (targeted panels, WES, WGS, RNA-seq, and scRNA-seq) for PF/IPF diagnosis, highlighting their advantages, limitations, cost, diagnostic yield, and current clinical applicability.

Technology	Detection	Key Applications in PF/IPF	Advantages	Limitations	Clinical Status
Targeted Gene Panels	Predefined set of genes (e.g., TERT, SFTPC, MUC5B)	Diagnostic confirmation in suspected FPF; identifying molecular subtypes; prognostic stratification	Cost-effective; rapid turnaround; high depth of coverage; easier interpretation	Limited to known genes; may miss novel or unexpected variants	Clinical use in expert centres for selected cases
Whole-Exome Sequencing (WES)	Protein-coding regions of all genes	Discovery of rare variants in known and novel genes; research in FPF; complex cases with negative panel	Broader coverage than panels; can identify novel genes	Higher cost; incidental findings; interpretation challenges	Translational; limited to research or complex cases
Whole-Genome Sequencing (WGS)	Entire genome (coding and non-coding)	Comprehensive variant detection; research on structural variants and non-coding regions	Most comprehensive; detects non-coding, structural variants	Highest cost; data storage/analysis; interpretation complexity	Research; not yet for routine clinical use
RNA Sequencing (RNA-seq)	Gene expression levels; splicing; non-coding RNAs	Pathway analysis; biomarker discovery; molecular phenotyping	Functional insights; can validate genomic findings; reveals cellular heterogeneity	Requires high-quality RNA; complex analysis; not standardised	Research; developing potential as diagnostic classifier
Single-cell RNA-seq (scRNA-seq)	Gene expression at individual cell resolution	Mapping cellular heterogeneity; identifying rare cell populations; understanding fibrotic niche	Highest resolution of tissue biology	Very high cost; complex analysis; difficult to standardise	Research; fundamental for discovery

**Table 4:** Key published NGS and GWAS studies in pulmonary fibrosis (selected high-impact, large-cohort studies). Includes study design, sample size, platform, main findings, clinical relevance, and limitations.

Study	Study Type	Sample Size	Sequencing Platform/Technology	Key Genetic/Transcriptomic Findings	Clinical Relevance	Limitations
[8]	Case–control GWAS	1610 IPF cases, 2477 controls	SNP microarray	MUC5B promoter variant (rs35705950) confers strong IPF risk (OR ~6–9)	First major common variant; changed understanding of IPF genetics	Non-NGS; European ancestry only
[30]	GWAS	1616 IPF cases, 4683 controls	Illumina arrays	Multiple susceptibility loci (TERT, RTEL1, MUC5B, etc.)	Established polygenic architecture of IPF	Non-NGS; limited functional validation
[110]	Candidate gene sequencing	1226 IPF cases, 1286 controls	Targeted NGS of telomerase genes	Rare protein-altering variants in TERT, TERC, PARN associated with IPF; MUC5B interacts	Showed rare variants contribute to sporadic IPF	Targeted panel only; limited to telomerase genes
[6]	Whole-exome sequencing	1548 FPF cases, 1991 familial controls	WES	Pathogenic variants in telomere-related (27%) and surfactant-related (5%) genes; novel genes (SPATA6, TP63)	Defined genetic landscape of FPF; diagnostic yield quantified	Retrospective; selection bias toward families |
[17]	Targeted NGS + telomere length	1022 PF patients (multi-ethnic)	Targeted panel (telomere/surfactant genes)	Telomere-related variants in 12%; shorter telomeres associated with mortality across races	Demonstrated racial differences in telomere length and outcomes	No WGS; telomere length not directly sequenced
[35]	Expert consensus + cohort review	1067 FPF cases	Various (panel/WES)	Summary of diagnostic yield: ~25–30% in FPF, ~5–10% in sporadic	Provided clinical recommendations for genetic testing	Not a primary research study; consensus-based
[97]	GWAS of longitudinal lung function	2115 IPF cases	GWAS array	PCSK6 and other variants associated with FVC decline and survival	First large-scale GWAS for disease progression	Non-NGS; need functional studies
[98]	Exome array + validation	1482 IPF cases	Exome array (targeted)	PCSK6 variant associated with survival; replicated in multiple cohorts	Potential prognostic biomarker	Not full WES/WGS; mechanism unclear
[100]	Transcriptomic (RNA-seq)	263 IPF patients (discovery + validation)	RNA-seq of peripheral blood	52-gene signature predictive of transplant-free survival	First validated blood-based prognostic signature for IPF	Single-centre discovery; requires qPCR implementation
[99]	Multi-omics (RNA-seq + proteomics)	114 IPF patients	RNA-seq + proteomics	Identified two molecular endotypes with different progression rates	Demonstrates feasibility of molecular subtyping	Small sample size; validation needed
[79]	Whole-exome sequencing	142 Chinese IPF cases, 255 controls	WES	Novel susceptibility genes (DSP, DYNCIH1, etc.) and pathway enrichment	First WES in Chinese IPF population	Modest sample size; replication needed
[33]	Targeted NGS panel	624 FPF probands	Custom 150-gene panel	Pathogenic variants in 29% of FPF; telomere genes most common	Comprehensive panel for clinical FPF testing	No functional validation; VUS rate not reported

## Data Availability

No new data were created or analyzed in this study. Data sharing is not applicable to this article.
